# PICK1 Genetic Variation and Cognitive Function in Patients with Schizophrenia

**DOI:** 10.1038/s41598-017-01975-y

**Published:** 2017-05-15

**Authors:** Yi-Ting Chen, Chieh-Hsin Lin, Chiung-Hsien Huang, Wen-Miin Liang, Hsien-Yuan Lane

**Affiliations:** 1 Graduate Institute of Biomedical Sciences, China Medical University, Taichung, Taiwan; 2 Department of Psychiatry & Brain Disease Research Center, China Medical University Hospital, Taichung, Taiwan; 3 Department of Psychiatry, Chang Gung Memorial Hospital, Kaohsiung, Taiwan; 4 Center for General Education, Cheng Shiu University, Kaohsiung, Taiwan; 5 Graduate Institute of Biostatistics, School of Public Health, China Medical University, Taichung, Taiwan

## Abstract

The gene of protein interacting with C kinase 1 alpha (PICK1) has been implicated in schizophrenia, nevertheless, conflicting results existed. However, its role in cognitive function remains unclear. Besides, cognitive deficits impair the long-term outcome. We explored whether the polymorphisms of PICK1 (rs2076369, rs3952) affected cognitive functions in schizophrenic patients. We analyzed 302 patients and tested the differences of cognitive functions, clinical symptoms between genetic groups. We also used general linear model to analyze the effect of PICK1 genetic polymorphisms on cognitive functions. After adjustment for gender, age, education, the patients with rs2076369 G/T genotype showed better performance than T/T homozygotes in the summary score, global composite score, neurocognitive composite score, category fluency subtest, WAIS-III-Digit Symbol Coding subtest, working memory, WMS-III-Spatial Span (backward) subtest, MSCEIT-managing emotions branch (p = 0.038, 0.025, 0.046, 0.036, 0.025, 0.027, 0.035, 0.028, respectively). G/G homozygotes performed better than T/T in category fluency subtest (p = 0.049). A/A homozygotes of rs3952 performed better than G/G in trail making A subtest (p = 0.048). To our knowledge, this is the first study to indicate that PICK1 polymorphisms may associate with cognitive functions in schizophrenic patients. Further replication studies in healthy controls or other ethnic groups are warranted.

## Introduction

In the glutamate system, better known ionotropic receptors are the N-methyl-D-aspartate (NMDA) receptor and the α-amino-3-hydroxy-5-methyl-4-isoxazolepropionic acid (AMPA) receptor. Both of them play pivotal roles in neurodevelopment and cognition.

D-serine is synthesized from L-serine through serine racemase (SR) and is degraded through D-amino acid oxidase (DAAO)^1^. The NMDA receptor is vital for synaptic plasticity, however, AMPA receptor usually modulates rapid synaptic transmission^2^. Activation of AMPA receptor causes the exocytosis of D-serine from astrocytes^3^. Then, D-serine activates the NR1 subunit of NMDA receptor^3^.

PICK1, first identified by a yeast two-hybrid screen, is a PSD-95/Discs large/ZO-1 homologous (PDZ) domain protein, which interacts with protein kinase C-alpha (PKCα)^4^. SR binds to the PDZ domain of PICK1^5^. Furthermore, PICK1 colocalizes with AMPA receptors at excitatory synapses^6^, induces AMPA receptor synaptic activity with its internalization and down-regulation^3^. Phosphorylation of AMPA receptors by PKCα bound to PICK1 causes the activation of NMDA receptor via D-serine and glutamate^7^. Additionally, PICK1 may involve in neurological disorders such as schizophrenia, via its interaction with subunit 2 of AMPA receptor (GluA2) and its influence on the excitatory synapse activity^8^.

NMDA receptor blockade induces behavioral and cognitive inflexibility^9^. Trafficking of AMPA receptors also modulates synaptic plasticity through long-term depression (LTD) and long-term potentiation (LTP), thereby influencing learning and memory^10, 11^. PICK1 structure modified by calcium is important for AMPA trafficking^12^ and the underlying mediation of hippocampal synaptic plasticity driven by NMDA receptor-dependent LTD^12^. PICK1 plays a crucial role in NMDA-induced AMPA receptor internalization and LTD by inhibition of Arp2/3-mediated actin polymerization; meanwhile, actin dynamics and synaptic function also modulate PICK1 via the regulator, Arf1^13^. Furthermore, PICK1 also mediates LTP by transient transformation of synaptic AMPA receptors during its trafficking^14^. In summary, LTD and/or LTP of synaptic AMPA receptors during trafficking are important for synaptic plasticity, which is critical for memory. In addition, genetic deletion of PICK1 exerts regulatory mechanism in hippocampal synaptic plasticity^11, 15^ and inhibitory avoidance learning^11^. PICK1 not only affects synaptic plasticity but also alters the neuronal architecture^16^. Therefore, NMDA receptors and AMPA receptors interrelate to PICK1.

There were inconsistent findings about the two single nucleotide polymorphisms (SNPs) of PICK1 (rs3952 and rs2076369) and schizophrenia. The study among Han Chinese showed an association between an SNP, rs3952, in intron 3 of PICK1, and schizophrenia^17^. In two Japanese populations, they did not find a relationship between rs3952 and schizophrenia^5, 18^. There were various allele frequencies among the different groups. Different minor allele exists between Han Chinese and Japanese populations (A, G, respectively). In one Japanese population, they found an association between rs2076369, in intron 4 of PICK1, and schizophrenia^5^. Nevertheless, in another Japanese and the Bulgarian population, rs2076369 did not associate PICK1 with schizophrenia^18, 19^. One of the possible explanations may be the different diagnostic criteria while Fujii *et al*. used the international statistical classification of the disease, revision 10 (ICD-10), Hong *et al*., Ishiguro *et al*. and the Bulgarian group applied the Diagnostic and Statistical Manual of Mental Status (DSM)^5, 17–19^. Another possible explanation may be resulted from how long the schizophrenia diagnosis maintained, while only Fujii *et al*. described that the schizophrenic diagnosis maintained unchanged at least 3 years^5, 17–19^. In addition, the Bulgarian group recruited patients with schizophrenia or schizoaffective disorder^19^. According to the concepts of “modifier genes”, we proposed that PICK1 may have an influence on the cognitive presentations only or have the combined effects of susceptibility and cognitive presentations of schizophrenia^20^.

Positive symptoms, negative symptoms, and cognitive dysfunction are the main manifestations of schizophrenia while cognitive impairment has the long-term influence on the functional outcome^21^. Moreover, cognitive dysfunction has been regarded as a candidate of endophenotype of schizophrenia^22^. On the other hand, the pathophysiology of schizophrenia has the compelling association with NMDA hypofunction^23^. Furthermore, N-methylglycine (sarcosine), known as endogenous glycine transporter-1 inhibitor (enhanced glycine acting on the subunit 1 of NMDA receptor), has the augmentative effect on the cognitive symptoms of chronic schizophrenia in our previous study^24^. Besides, NMDA receptors and AMPA receptors interrelate to PICK1. In addition, the relationship between PICK1 and cognitive function remains unclear. Therefore, we focus on the association of PICK1 with cognitive function.

The PDZ domain of PICK1 and its encoding gene bind to SR. SR transforms L-serine to D-serine. The AMPA receptor activates the exocytosis of D-serine. D-serine acts on NMDA receptor subunit 1. The NMDA hypofunction relates to cognitive dysfunction in schizophrenia. We, therefore, hypothesized that the related tendency between the two SNPs (rs3952 and rs2076369) in the PICK1 gene and cognitive dysfunction in schizophrenic patients. This study aimed to explore the association between genetic polymorphism of PICK1 and cognitive functions in schizophrenic patients.

## Results

### Demographic data in schizophrenic patients

We enrolled 310 patients with chronic stable schizophrenia and genotyped two SNPs (rs2076369, rs3952) of the PICK1 gene. However, genotypes of 8 of them were unavailable using the laboratory method. Therefore, 302 patients were eligible for analysis, with a mean age ± SD (38.1 ± 9.4 years) and a mean education level ± SD (10.9 ± 2.4 years). Their mean age at illness onset ± SD was 23.2 ± 6.7 years old, the mean illness duration ± SD was 186.0 ± 248.6 months, the mean dose (shown as chlorpromazine equivalent) of the antipsychotics they used ± SD was 529.8 ± 490.4 mg/day.

There were no significant differences between different genotypes of PICK1 in the demographic data (gender, age, education, age at illness onset, illness duration, antipsychotic dosages) (Table 1).

**Table 1:** Demographics of schizophrenic patients with different genotypes of PICK1.

Clinical characteristics	rs2076369				rs3952			
	TT	GT	GG	p	AA	AG	GG	p
Male/Female	13/8	69/51	97/64	0.869	59/38	91/54	29/31	0.149
Age, year	37.10 (10.387)	39.46 (9.390)	37.25 (9.181)	0.130	38.62 (9.401)	38.68 (9.195)	35.95 (9.640)	0.136
Education, year	10.38 (1.564)	10.85 (2.523)	10.99 (2.366)	0.520	10.91 (2.463)	10.91 (2.429)	10.82 (2.167)	0.998
Age at illness onset, year	21.76 (7.245)	24.08 (7.458)	22.70 (5.849)	0.198	23.40 (6.806)	23.41 (6.990)	22.27 (5.480)	0.734
Illness duration, months	184.00 (108.804)	209.05 (373.722)	169.07 (101.021)	0.689	174.27 (106.674)	203.19 (341.497)	163.42 (103.113)	0.486
Antipsychotic dose, mg/day*	527.50 (462.425)	568.54 (643.838)	501.23 (339.773)	0.556	553.26 (716.057)	532.32 (361.405)	485.78 (266.378)	0.355

All data were presented by mean (standard deviation).

Chi-Square test for categorical variables (gender); according to normality of data, Kruskal-Wallis test for continuous variables (except age which was normal distribution, so we used ANOVA test).

*Antipsychotic dose, presented by chlopromazine equivalent dose.

### The association between different genotypes of PICK1 genetic polymorphism and clinical characteristics in schizophrenic patients

Their mean PANSS total score ± SD was 85.0 ± 13.2, mean PANSS-positive subscale score ± SD was 19.9 ± 4.6, mean PANSS-negative subscale score ± SD was 23.8 ± 5.1, and mean SANS 20-total score ± SD was 50.9 ± 15.9.

There were no significant associations between SNPs (rs2076369, rs3952) and clinical symptoms, including PANSS total score, PANSS-positive score, PANSS-negative score, and SANS 20-total score (Table 2).

**Table 2:** Association between genotypes of PICK1 and clinical symptoms in schizophrenic patients.

Cognitive domain	rs2076369				rs3952			
	TT	GT	GG	p	AA	AG	GG	p
PANSS total	89.86 (17.109)	83.78 (13.289)	85.23 (12.510)	0.439	83.91 (14.433)	86.20 (13.316)	83.75 (10.608)	0.552
PANSS^a^-positive subscale	20. 86 (5.651)	19.39 (4.582)	20.11 (4.375)	0.504	19.28 (4.974)	20.20 (4.448)	20.07 (4.057)	0.265
PANSS-negative subscale	26.52 (7.097)	23.73 (5.174)	23.52 (4.709)	0.119	24.24 (5.882)	24.01 (5.085)	22.62 (3.580)	0.125
SANS^b^ 20-total	55.67 (18.315)	50.57 (16.209)	50.43 (15.382)	0.357	51.09 (16.350)	51.46 (16.713)	48.98 (13.149)	0.590

All data were presented by mean (standard deviation).

According to normality of data, Analysis of Variance ﻿(ANOVA) was used for SANS 20-total and Kruskal-Wallis test for other items.

^a^PANSS = Positive and Negative Syndrome Scale.

^b^SANS = Scale for the Assessment of Negative Symptoms.

### Distribution of allele frequency in PICK1 with different SNPs among schizophrenic patients

Among schizophrenic patients of rs2076369, 161 had G/G genotypes, 120 had G/T genotypes and 21 had T/T genotypes; among those of rs3952, 97 had A/A genotypes, 145 had A/G and the other 60 had G/G genotypes. The distribution of three genotypes of rs2076369 in PICK1 and that of rs3952 in PICK1 did not deviate from the Hardy-Weinberg equilibrium (p = 0.83, 0.66, respectively).

Regarding the allele distribution, the minor allele frequency of rs2076369 (T allele: 26.8%) in this study was lower than that in Japanese populations (46.2%, 41.4%, respectively)^5, 18^. In terms of rs3952, the minor allele frequency in this study (G allele: 43.9%) was higher than that in both Japanese populations (32.0%, 39.0%, respectively)^5, 18^. However, the minor allele of rs3952 in another Han Chinese population was A allele^17^ (Table 3).

**Table 3:** PICK1 allele distribution in chronic schizophrenic patients of our study (n = 302), Fujii *et al*.^5^ (n = 200), Hong *et al*.^17^ (n = 225), and Ishiguro *et al*.^18^ (n = 1851).

Research group	rs2076369				rs3952			
	TT(%)	GT(%)	GG(%)	MAF(Allele)	AA(%)	AG(%)	GG(%)	MAF(Allele)
Our study	6.95	39.74	53.31	0.268 (T)	32.12	48.01	19.87	0.439 (G)
Fujii *et al*.	N/A	N/A	N/A	0.462 (T)	N/A	N/A	N/A	0.320 (G)
Hong *et al*.	N/A	N/A	N/A	N/A	9.8	51.1	39.1	0.353 (A)
Ishiguro *et al*.	17.7	47.4	34.9	0.414 (T)	46.3	42.0	11.7	0.390 (G)

**MAF** = Minor allele frequency, **N/A** = no data applicable.

### The association between PICK1 genetic variations and cognitive function in schizophrenic patients

Since in this paper, we mainly assessed the effect of genotypes on the different measures of cognitive functions, we, therefore, chose to clarify the effect of genotypes within each polymorphism in Table 4. All the data were standardized to T scores^25^. The cognitive function among 3 genotypes of rs2076369 had significant differences in working memory and WMS-III-spatial span (backward) subtest (p = 0.024 and 0.030, respectively) (Table 4). Therefore, we applied post-hoc analysis with Bonferroni test to examine the between-genotype differences. In the working memory, those with G/T also had higher scores (better performance) than T/T homozygotes (51.5 ± 10.4 vs. 45.6 ± 9.8, p = 0.034), however, G/T had better performance than G/G without statistical significance (51.5 ± 10.5 vs. 49.5 ± 9.6, p = 0.252). In the WMS-III-spatial span (backward) subtest, the patients with the G/T genotype had higher scores (better performance) than T/T homozygotes (51.5 ± 10.5 vs. 45.9 ± 10.7, p = 0.049), on the contrast, G/T had the tendency of better performance than G/G without statistical significance (51.5 ± 10.5 vs. 49.4 ± 9.4, p = 0.226). In summary, PICK1 genetic variation (rs2076369) partially associated with cognitive function.

**Table 4:** The association between genotypes of PICK1 and cognitive function in schizophrenic patients.

Cognitive domain	rs2076369				rs3952			
	TT	GT	GG	p	AA	AG	GG	p
Summary score^a^	45.9 (9.5)	51.0 (10.3)	49.8 (9.8)	0.089	50.3 (10.3)	50.3 (9.6)	48.9 (10.4)	0.607
Global composite score^b^	45.5 (9.4)	51.0 (10.3)	49.8 (9.8)	0.063	50.2 (10.4)	50.3 (9.6)	49.0 (10.4)	0.664
Neurocognitive composite score^c^	45.9 (9.3)	51.0 (10.3)	49.8 (9.7)	0.090	50.2 (10.1)	50.4 (9.7)	48.8 (10.6)	0.556
1. Speed of processing^d^	45.6 (7.6)	51.1 (10.6)	49.8 (9.7)	0.059	49.6 (9.6)	50.6 (10.3)	49.3 (10.0)	0.650
Category Fluency	45.5 (8.6)	50.8 (10.0)	50.0 (10.1)	0.080	49.9 (9.8)	50.6 (9.6)	48.7 (11.2)	0.484
Trail Making A	45.6 (10.3)	50.2 (10.3)	49.9 (9.8)	0.952	48.7 (9.7)	50.5 (10.2)	50.9 (1.0)	0.286
WAIS^e^-III,Digit Symbol Coding	45.8 (6.7)	51.3 (10.9)	49.6 (9.5)	0.051	50.6 (10.0)	50.1 (10.1)	48.9 (9.7)	0.597
2. Attention: CPT-IP^f^	49.9 (9.6)	49.1 (1.0)	50.7 (10.1)	0.440	50.8 (10.6)	49.6 (9.4)	49.8 (10. 7)	0.655
3. Working Memory^g^	45.6 (9.8)	51.5 (10.4)	49.5 (9.6)	0.024	50.0 (10.0)	50. 5 (9.8)	48. 8 (10.5)	0.517
Backward digit span	46.5 (8.8)	51.1 (10.4)	49.7 (9.8)	0.128	50.0 (10.0)	50.6 (9.8)	48.6 (10.4)	0.446
WMS^h^-III-Spatial Span (backward)	45.9 (10.7)	51.5 (10.5)	49.4 (9.4)	0.030	49.9 (10.2)	50.4 (10.1)	49.3 (9.5)	0.782
4. Verbal learning and memory: WMS-III, word listing	48.7 (10.1)	50.5 (9.8)	49.8 (10.2)	0.706	50.8 (1.0)	49.6 (9.9)	49.6 (10.4)	0.604
5. Visual learning and memory: WMS-III, visual reproduction	47.3 (8.5)	50.9 (10.9)	49.7 (9.5)	0.267	50.5 (1.0)	50.3 (10.3)	48.5 (9.3)	0.418
6. Reasoning and problem solving: WISC^i^-III, Maze	48.8 (10.8)	50.0 (9.9)	50.1 (10.1)	0.837	50.0 (9.4)	50.5 (10.3)	48.8 (10.4)	0.542
7. Social cognition: The managing emotions branch of MSCEIT^j^.	46.0 (9.0)	50.6 (9.5)	50.1 (10.4)	0.154	50.0 (10.6)	49.8 (9.2)	50.7 (10.9)	0.845

All data were standardized to T scores and presented by mean (standard deviation).

According to normality of data, ANOVA was used for all scales. If the result was significant, the post-hoc analysis with Bonferroni test was applied to examine the between-genotype differences (data was shown in the results section).

^a^ **Summary score:** principal component analysis with varimax rotation showed the 7 domains of cognitive tests can be reduced to one dimension and we calculated the estimated summary score.

^b^ **Global composite score**: an overall composite T scores that included all 7 domains was calculated by standardizing the sum of T scores.

^c^ **Neurocognitive composite score**: an overall composite T scores that included all 6 neurocognitive domains, excluding social cognition, was calculated by standardizing the sum of T scores.

^d^ **Speed of processing**: an overall composite T scores that included all 3 domains (Category fluency, Trail making A, WAIS-III Digit Symbol- coding) was calculated by standardizing the sum of T scores.

^e^ **WAIS-III**: Wechsler adult intelligence scale-Third Edition.

^f^ **CPT-IP**: Continuous Performance Test-Identical Pairs (only showed CPT1- d′ value: the d′ value in the first session of CPT).

^g^ **Working memory**: an overall composite T scores that included all 2 domains (Backward digit span, WMS-III, Spatial span) was calculated by standardizing the sum of T scores.

^h^ **WMS-III**: Wechsler memory scale-Third Edition.

^I^ **WISC-III**: Wechsler intelligence scale for children-Third Edition.

^j^ **MSCEIT:** Mayer-Salovey-Caruso Emotional Intelligence Test.

We afterward performed Haploview 4.2^26^ to estimate the pairwise linkage disequilibrium (LD) and measure the r^2^ and D´ values. The pair of SNPs showed that the r^2^ and D´ values were 0.287, 1.0, respectively. Therefore, the LD was assumed. Thereafter, in Table 5, we ran our model to fit both polymorphisms in the same model to correct the influence by the other polymorphism. We used general linear model (GLM) to control gender, age, education and all the data were standardized to T scores. The data showed that the patients with the G/T genotype of rs2076369 performed better than the T/T homozygotes in the summary score, global composite score, neurocognitive composite score, category fluency subtest, WAIS-III-Digit Symbol Coding subtest, working memory, WMS-III-Spatial Span (backward) subtest, MSCEIT-managing emotions branch (p = 0.038, 0.025, 0.046, 0.036, 0.025, 0.027, 0.035, 0.028, respectively) (Table 5).

**Table 5:** Multiple regression model analysis of the effects of genetic polymorphism on cognitive function in schizophrenic patients by general linear model.

Cognitive domain		rs2076369			rs3952		
		GT *vs* TT	GT *vs* GG	TT *vs* GG	AG *vs* GG	AG *vs* AA	GG *vs* AA
Summary score	β	5.0	0.8	−4.3	−0.01	−1.1	−1.1
	95% CI	(0.3, 9.7)	(−1.8, 3.3)	(−9.1, 0.6)	(−3.2, 3.2)	(−3.7, 1.6)	(−4.7, 2.5)
	p	0.038	0.565	0.087	0.997	0.428	0.562
Global composite Score	β	5.4	0.9	−4.4	−0.2	−1.0	−0.8
	95% CI	(0.7, 10.1)	(−1.6, 3.5)	(−9.3, 0.4)	(−3.4, 2.9)	(−3.6, 1.7)	(−4.4, 2.9)
	p	0.025	0.470	0.074	0.892	0.473	0.682
Neuro-cognitive composite score	β	4.8	0.7	−4.1	0.2	−0.8	−1.0
	95% CI	(0.1, 9.5)	(−1.8, 3.3)	(−9.0, 0.8)	(−3.0, 3.4)	(−3.5, 1.8)	(−4.6, 2.6)
	p	0.046	0.569	0.102	0.914	0.540	0.585
1. Speed of processing	β	4.8	1.2	−3.6	−0.3	0.1	0.3
	95% CI	(−0.003, 9.7)	(−1.4, 3.8)	(−8.6, 1.4)	(−3.5, 3.0)	(−2.6, 2.8)	(−3.4, 4.1)
	p	0.050	0.357	0.157	0.877	0.952	0.858
Category Fluency	β	5.3	0.2	−5.1	1.3	−0.5	−1.8
	95% CI	(0.4, 10.2)	(−2.5, 2.8)	(−10.2, −0.03)	(−2.0, 4.6)	(−3.3, 2.3)	(−5.6, 2.0)
	p	0.036	0.910	0.049	0.444	0.738	0.359
Trail Making A	β	−0.7	1.3	2.0	−1.7	2.1	3.8
	95% CI	(−5.7, 4.2)	(−1.4, 4.0)	(−3.1, 7.2)	(−5.0, 1.7)	(−0.7, 4.9)	(0.03, 7.6)
	p	0.773	0.334	0.433	0.321	0.132	0.048
WAIS-III,Digit Symbol Coding	β	5.5	1.1	−4.4	−0.1	−1.5	−1.4
	95% CI	(0.7, 10.2)	(−1.5, 3.7)	(−9.3, 0.5)	(−3.3, 3.1)	(−4.2, 1.2)	(−5.0, 2.3)
	p	0.025	0.413	0.080	0.933	0.273	0.464
2. Attention: CPT-IP	β	0.3	−1.7	−2.0	0.2	−1.7	−1.9
	95% CI	(−4.6, 5.2)	(−4.4, 1.0)	(−7.1, 3.1)	(−3.1, 3.5)	(−4.4, 1.1)	(−5.6, 1.9)
	p	0.901	0.206	0.434	0.899	0.238	0.328
3. Working Memory	β	5.2	1.8	−3.4	−0.7	−0.3	0.4
	95% CI	(0.6, 9.7)	(−0.7, 4.2)	(−8.1, 1.3)	(−3.7, 2.4)	(−2.9, 2.3)	(−3.1, 3.8)
	p	0.027	0.163	0.155	0.681	0.825	0.843
Backward digit span	β	3.9	0.9	−3.0	0.4	−0.2	−0.6
	95% CI	(−0.9, 8.6)	(−1.7, 3.4)	(−7.9, 1.9)	(−2.8, 3.6)	(−2.9, 2.5)	(−4.2, 3.1)
	p	0.111	0.517	0.228	0. 821	0.879	0.757
WMS-III-Spatial Span (backward)	β	5.0	2.1	−2.8	−1.5	−0.3	1.2
	95% CI	(0.4, 9.6)	(−0.4, 4.6)	(−7.6, 2.0)	(−4.6, 1.7)	(−2.9, 2.3)	(−2.4, 4.7)
	p	0.035	0.092	0.246	0.356	0.829	0.515
4. Verbal Learning and memory: WMS-III, word listing	β	1.3	−0.3	−1.6	−0.7	−1.6	−0.9
	95% CI	(−3.3, 5.8)	(−2.8, 2.1)	(−6.3, 3.1)	(−3.7, 2.4)	(−4.1, 1.0)	(−4.4, 2.6)
	p	0.583	0.801	0.507	0.660	0.229	0.618
5. Visual learning and memory: WMS-III, visual reproduction	β	3.6	0.2	−3.3	1.4	−1.0	−2.4
	95% CI	(−1.3, 8.5)	(−2.4, 2.9)	(−8.4, 1.7)	(−1.9, 4.7)	(−3.8, 1.8)	(−6.2, 1.3)
	p	0.153	0.859	0.197	0.392	0.483	0.205
6. Reasoning and problem solving: WISC-III, Maze	β	2.0	0.3	−1.8	1.6	0.1	−1.6
	95% CI	(−2.6, 6.6)	(−2.2, 2.7)	(−6.5, 3.0)	(−1.5, 4.7)	(−2.5, 2.6)	(−5.1, 2.0)
	p	0.388	0.845	0.464	0.307	0.972	0.384
7. Social cognition: The Managing emotions branch of MSCEIT	β	5.3	1.5	−3.8	−2.2	−1.2	1.1
	95% CI	(0.6, 10.0)	(−1.1, 4.0)	(−8.7, 1.0)	(−5.4, 1.0)	(−3.8, 1.5)	(−2.6, 4.7)
	p	0.028	0.262	0.123	0.171	0.393	0.567

All data were standardized to T scores.

β = Estimated Coefficient, 95% CI = 95% confidence interval.

P-value was based on the multiple regression model analysis of cognitive function between genetic groups by General linear model (GLM). We ran our model to fit both polymorphisms in the same model to correct the influence by the other polymorphism; in addition, we also included age, gender and years of education in the model.

The results of G/T heterozygotes showed a trend of better performance than the G/G homozygotes in all the domains except on the Trail Making A subtest (the higher scores indicated the poorer performance), CPT-IP (the d′ value in the first session of CPT) and WMS-III-word listing subtest which showed G/G had better performance than G/T, but they did not reach statistical significance. G/G homozygotes tended to perform better than T/T in category fluency subtest (p = 0.049); nevertheless, the difference did not reach statistical significance in the other domains (Table 5).

The cognitive function among 3 genotypes of rs3952 had no significant differences (Table 4). However, after controlling gender, age, and education, A/A homozygotes performed better than G/G in trail making A subtest (p = 0.048) (Table 5).

Principal component analysis showed that the 7 domains of cognitive tests can be reduced to one dimension. We, therefore, calculated the estimated summary score and re-analyzed it as shown in Tables 4 and 5. The results were similar to the global composite score.

## Discussion

Our findings disclosed that patients with G/T heterozygote of PICK1 (rs2076369) performed better than those with T/T homozygotes in the summary score, global composite score, neurocognitive composite score, category fluency subtest, WAIS-III-Digit Symbol Coding subtest, working memory, WMS-III-Spatial Span (backward) subtest, MSCEIT-managing emotions branch (p = 0.038, 0.025, 0.046, 0.036, 0.025, 0.027, 0.035, 0.028, respectively) (Table 5). On the other hand, there was not much difference between the two homozygotes. It was surprising. If T was the risk allele for the cognition, then we would expect that the performance would be better in no T allele (G/G genotype) than two copies of T alleles. One phenomenon of “molecular heterosis”, which occurs when subjects heterozygous for a specific genetic polymorphism display a greater or lesser effect for quantitative or dichotomous trait than subjects homozygous for either allele^27, 28^. Interestingly, our report indicated that G/T heterozygotes did not perform much better than G/G homozygotes and there was also no significant difference between T/T homozygotes and G/G homozygotes. This might be another example of heterosis^29^. So, the homozygous expression of rs2076369 with PICK1 (G/G and T/T) may have an excessive or insufficient impact on the neuronal trafficking, which may relate to the cognitive performance. On the other hand, our reports could not be completely explained by this theory^27, 28^ because G/T heterozygotes did not perform better than G/G homozygotes. Furthermore, in the central nervous system, the effect of dopamine receptor D2 gene on the striatal D2 receptor density also has a similar expression explained by molecular heterosis^28^. Besides, D2 receptor could modulate various brain functions, such as learning, working memory, and synaptic transmission^30^. In addition, PICK1 is colocalized with the dopamine transporter^31^. Dopamine 1 receptor agonists may benefit for ketamine-induced working memory anomalies in the schizophrenic animal model^32^. Together with those reports, we proposed that dopaminergic system and its genetic polymorphisms may interact with the PICK1 gene. In the future, studies with larger samples are needed to elucidate these issues.

Schizophrenic patients have deficient category fluency^33^. Poor performance in the category fluency subtest, which reflects the speed of processing, is also found in dementia^34^. Category fluency subtest could also be used to discriminate Chinese healthy and the elderly with Alzheimer’s disease at a cutoff score of 24/25 with the sensitivity of 86.8% and specificity of 93.4%^35^. Furthermore, either using category fluency subtest alone or the combination of category fluency and letter fluency subtest could detect mild Alzheimer’s disease^36^. Besides, whether genetic variation of PICK1 also affects the category fluency of patients with dementia of the Alzheimer type deserves studies too. According to the findings of multi-channel near-infrared spectroscopy, schizophrenic patients have decreased cortical activation over the left prefrontal region during the Verbal Fluency Test, including letter and category version, compared to healthy controls^37^. Therefore, PICK1 may exert its impact on the category fluency subtest via left frontal lobe and more studies wait to be investigated.

Meta-analysis presents that the impairment of digit symbol coding not only serves as cardinal cognitive deficits of the disease and the functional outcome in schizophrenic patients, but also a predictor for high-risk group^38^. Another study implicates that the memory impairment may explain the slowed processing speed tapped by digit symbol-coding subtest in schizophrenic patients^39^. Thus, the underlying mechanism of how the PICK1 affects the brain pathway and the digit symbol coding subtest is worthy to be detected.

SNP (rs2076369) of PICK1 had some association with working memory in our schizophrenic patients. Additionally, the comparison between first-episode antipsychotic-naïve schizophrenic patients and healthy controls shows significant impairments in working memory^40^. Namely, PICK1 may involve in working memory. Furthermore, the frontal gray matter density reduction may reflect the disconnection of the frontotemporal area and working memory deficit^41^. Accordingly, PICK1 may adjust working memory through the frontotemporal region. Future investigations aim to explore the influences of PICK1 between cognitive dysfunction and brain pathology of schizophrenia are necessary.

In accordance with our finding that the PICK1 genetic variation (rs2076369) in schizophrenic patients may associate with spatial working memory, review articles also show that schizophrenic patients have more severe dysfunction of spatial working memory compared to healthy controls^42^. Apart from, the results of knock-in mice lacking the PDZ-ligand motif of mGluR7a (metabotropic glutamate receptor 7a), with disruption of PICK1-mGluR7a interaction, also demonstrate spatial working memory deficits^43^. Furthermore, illustrating in other mouse models, PICK1 not only interacts with GluA2 but also affects the synapto-depressive effect of amyloid beta (Aβ) and plays a crucial role in Alzheimer’s disease^44^. Besides, LTD in the rat hippocampus relates to the interaction between phosphorylated tau and glycogen synthase kinase-3 (GSK-3)^45^. At the same time, PICK1 is a substrate of GSK-3 and PICK1 phosphorylation regulates the GluA2-PICK1 interaction^46^. That is, PICK1 may modulate spatial working memory in schizophrenic patients, as well as in mouse models of Alzheimer’s disease. Whether PICK1 influences on Alzheimer’s disease of human beings deserves to be deeply studied.

Schizophrenic patients have the malfunction in response to emotional faces^47^. Besides, dysfunction of facial emotional recognition involves precentral and inferior prefrontal area in schizophrenic patients compared to healthy controls^48^. Whether PICK1 also takes a part in the management of emotions via frontal area in schizophrenic patients remains to be studied.

The minor allele frequency of rs2076369 (T allele) in the current study was lower than that in the two Japanese populations (Table 3)^5, 18^. One of the possible explanations may be the different ethnicity. On the other hand, the minor allele frequency of rs3952 in the current study (G allele) was a bit higher than that in the two Japanese groups (Table 3)^5, 18^. The allele frequency fluctuation may stem from the changes in population compositions, and may not be related to the functions of the variant. In contrast, the minor allele of rs3952 in another Han Chinese population was A allele (Table 3)^17^. Nevertheless, the tested variants are intronic polymorphisms; the results might indicate its linkage disequilibrium with other functional polymorphisms. The inconsistent findings may actually reflect the variable linkage disequilibrium patterns across populations. Therefore, we used browser Ensembl GRCh37 of IGSR database (http://grch37.ensembl.org/index.html) in Southern Han Chinese population to search for the possible functional polymorphisms correlated with the two tested SNPs (see Supplementary Tables S1 and S2). Our data showed that the linkage disequilibrium between rs3952 and rs2076369 with r^2^ and D´ values were 0.287 and 1.0 which were similar to the results from the above-mentioned database which revealed 0.233 and 1.0. We need to do further investigations to elucidate the actual relationships between the 2 tested SNPs and other possible functional polymorphisms in the future.

Two limitations exist in this study. First, our findings could not explain the causality in the association between the genetic polymorphism and cognitive dysfunction. Concurrently, the cognitive dysfunction in schizophrenic patients could be influenced by many confounding factors, such as negative symptoms which strongly affect the neurocognition, social cognition, and functional outcome^49, 50^. Besides, there was no between-group difference in negative symptoms in the current study. So we did not control for this confounding factor in our analysis. Second, we did not enroll healthy individuals. Whether the finding can be significant after compared to the healthy volunteers or extrapolated to other races remains unknown.

In summary, this is the first study to show that PICK1 genetic polymorphisms (rs2076369, rs3952) may take a part in cognition. Further replication studies in healthy controls or other ethnic groups are warranted.

## Methods

This study was approved by the institutional review board (IRB) of the China Medical University Hospital and carried out in accordance with the Declaration of Helsinki. Written informed consent was obtained from each subject in line with the IRB’s guidelines.

### Participants

Three hundred and ten Han Taiwanese patients with schizophrenia aged from 18 to 65 years were recruited. Experienced research psychiatrists confirmed the diagnosis of schizophrenia using the Structured Clinical Interview for DSM-IV^51^.

All the enrolled patients fulfilled the DSM-IV diagnosis of schizophrenia and they remained symptomatic but clinically stable. Their antipsychotic doses were unchanged for at least 2 months. Patients with Axis I diagnosis other than schizophrenia or serious medical or neurological illness were excluded. All the enrolled patients had normal physical examinations, neurological examinations, and laboratory screening tests.

### Clinical assessments

We measured cognitive functions and clinical manifestations, including Positive and Negative Syndrome Scale (PANSS)^52^, Scale for Assessment of Negative Symptoms (SANS)^53^.

All the participants received clinical ratings by trained and experienced research psychiatrist.

### Measurements of cognitive function

Our cognitive battery, which were the same or similar to the tests from Measurement and Treatment Research to Improve Cognition in Schizophrenia (MATRICS), included 7 domains: (1) speed of processing: Category Fluency, Trail Making A^54^, and Wechsler adult intelligence scale-Third Edition (WAIS-III)-Digit Symbol-Coding^55^; (2) sustained attention: Continuous Performance Test-Identical Pairs (CPT-IP)^56^; (3) working memory: verbal: backward digit span^57^ and nonverbal: Wechsler memory scale-Third Edition (WMS-III)-Spatial Span (backward)^58^; (4) verbal learning and memory: WMS-III-word listing^58^; (5) visual learning and memory: WMS-III-visual reproduction^58^; (6) reasoning and problem solving: maze from Wechsler intelligence scale for children-Third Edition (WISC-III)^59^ and (7) social cognition: the managing emotions branch of Mayer-Salovey-Caruso Emotional Intelligence Test (MSCEIT)^60^. The Chinese version of the MSCEIT tasks was translated and back-translated from English to Mandarin Chinese with satisfactory reliability^61^, validity^61^ and applicability^61, 62^.

### Genotyping

To detect the −472G/T polymorphism (rs2076369) and −475A/G polymorphism (rs3952), DNA was isolated from the blood using the MasterPure DNA Purification Kit for Blood Version II (EPICENTRE, Madison, Wisconsin, USA) according to the protocol provided by the manufacturer. DNA concentration was determined by absorbance at 260 nM (ND-1000 UV-Vis spectrophotometer, Thermo Fisher Scientific Inc.). Before PCR reaction, DNA was diluted to a concentration of 50 ng/ul.

All SNP genotyping was performed using the Taqman SNP genotyping assay (ABI: Applied Biosystems Inc., Foster City, CA, USA). Standard DNA samples with known genotypes were used for quality control. The PCR reaction was conducted in the 10 μl reaction volume, containing 0.4 μl DNA sample, 5 μl PCR master mix, and 0.25 μl primer pairs and probes. A pre-incubation at 95 °C for 10 min was used to activate the Hot-Start DNA polymerase and denature DNA and was followed by 40 amplification cycles of 92 °C denaturation for 15 Sec; and 60 °C for 60 Sec. The probe fluorescence signal detection was performed using the ABI Prism 7500 Real-Time PCR System.

### Statistical analysis

We analyzed the data using the Statistical Package for the Social Sciences (SPSS) version 17. The deviation of the genotype counts from the Hardy–Weinberg equilibrium was tested by employing a Chi-Square goodness-of-fit test. We used Haploview 4.2^26^ to estimate the pairwise linkage disequilibrium (LD) and measure the r^2^ and D´ values. If the pair of SNPs had r^2^, D´ closer to 1, then the linkage disequilibrium was assumed. Scores of the cognitive tests were standardized to T scores^25^. The difference of demographics and clinical symptoms between genetic groups was tested by Chi-Square test, Analysis of Variance (ANOVA) or Kruskal-Wallis test. We assessed the effects of genotype on the cognitive function separately for each SNP. Since there were three genotypes for each SNP, we performed the one-way ANOVA to assess the effect of genotype on each scale of cognitive function. Furthermore, when the result was significant, we performed the Bonferroni correction method to do the pairwise comparisons among the three genotypes. We did not consider two analysis of SNP at the same time in Table 4. Since in this paper, we mainly assessed the effect of genotypes on the different measures of cognition functions, therefore, we chose to clarify the effect of genotypes within each polymorphism in Table 4. In Table 5, we tried to rerun our model to fit both polymorphisms in the same model to correct the influence by the other polymorphism.

We examined the correlation patterns among all cognitive tests which showed highly correlated pattern existed. Therefore, we performed principal component analysis with varimax rotation to determine how many dimensions could be extracted from multiple subscales of cognitive tests.

Kern *et al*. suggested that age, education and gender difference should be corrected in trials while using MATRICS Consensus Cognitive Battery (MCCB)^25^. In addition, we considered the illness duration, onset age, antipsychotic dosages as potential covariates, which may influence the cognitive performance of the patient’s prognosis. We used variance inflation factor (VIF) as the indicator to check whether the multi-collinearity existed among independent variables (gender, age, education, onset age, illness duration, antipsychotic dosages) in the multiple regression analysis. When the VIF value of one independent variable was more than 10, it usually indicated collinearity existed among the independent variable and the other ones. The VIF values of gender, age, education, illness duration, onset age, antipsychotic dosages in our study were all less than 2. Besides, we also searched for the independent variables by forward, backward and stepwise selection to examine whether the model was over-adjustment. The three methods suggested gender, age and education were suitable. Therefore, we chose gender, age and education as covariates.

There are many characteristics in the cognitive expression which could express the specific concept of what the developer of questionnaires really expected^63^. It may be rather difficult for patients to answer one global question and achieve the same target concept of what the developer of questionnaires really expect^63^. In another word, greater precision may be obtained using multiple related domains (items) instead of a single domain (item)^63^. Therefore, we demonstrated and analyzed each specific concept separately. However, the result from principal component analysis supported one target domain covered the full breadth of different complex concepts in our analysis. Therefore, we calculated the power under the above-mentioned consideration that the cognitive function was one global aspect. The power estimates for this study was performed using the SAS System (version 9.3; SAS Institute, Cary, NC). In assessing the association between 3 genotypes of rs2076369 and cognitive function, the sample sizes for GT, GG, and TT among 3 genotypes of rs2076369 were 120, 161 and 21. We assumed that each group had a standard deviation 9.6, which was estimated by the average value of standard deviations of the three groups in the global composite score. Assume that the effect of the difference of the means between the lowest group and the highest group was 6 and the mean of the middle group was their average, the power will reach 0.925 with type I error of 0.05 for the one-way ANOVA analysis. We further raised an example based on our data to demonstrate the power of our test. We assumed that the population means of the T scores of working memory for GT, GG, and TT were 51.5, 49.5, and 45.6. According to the above setting criterion, the overall power of the F-test using one-way ANOVA reached 0.878 and the power of detecting the difference between the GT and TT groups reached 0.928.

## Electronic supplementary material

Supplementary Table
