# Supplementary material for: PICK1 Genetic Variation and Cognitive Function in Patients with Schizophrenia
Source: Sci Rep. 2017 May 15;7:1889. doi: 10.1038/s41598-017-01975-y (PMC5432511; doi:10.1038/s41598-017-01975-y)
Supplement: Supplementary file 1 — Supplementary Table [file 41598_2017_1975_MOESM1_ESM.docx]

**PICK1 Genetic Variation and Cognitive Function in Patients with Schizophrenia**

Yi-Ting Chen^1,2^, Chieh-Hsin Lin^1,3,4^, Chiung- Hsien Huang^1,2^, Wen-Miin Liang^5^ & Hsien-Yuan Lane^1,2,^*

^1^ Graduate Institute of Biomedical Sciences, China Medical University, Taichung, Taiwan.

^2^ Department of Psychiatry, & Brain Disease Research Center, China Medical University Hospital, Taichung, Taiwan.

^3^ Department of Psychiatry, Chang Gung Memorial Hospital, Kaohsiung, Taiwan.

^4^ Center for General Education, Cheng Shiu University, Kaohsiung, Taiwan.

^5^ Graduate Institute of Biostatistics, School of Public Health, China Medical University, Taichung, Taiwan.

**Corresponding author at:**

Department of Psychiatry, China Medical University Hospital,

No.2, Yude Rd., North Dist., Taichung City 40447, Taiwan (R.O.C.)

TEL: +886 921 067 260;

Fax: +886 4 2236 1042

E-mail: hylane@gmail.com (H.-Y. Lane)

**Supplementary Table S1.**Possible functional polymorphisms which have linkage disequilibrium with rs3952: Pairwise r^2^ values and pairwise D' values for 22:38050591-38070590. Population: 1000GENOMES:phase_3:CHS

<http://asia.ensembl.org/Homo_sapiens/Export/Output/Location?_format=HTML;db=core;output=ld;pop1=1000GENOMES:phase_3:CHS;r=22:38050591-38070590;v=rs3952;vdb=variation;vf=3758>

| **Pairwise r^2^ values** | **SNP** |
| --- | --- |
| 0.080 | rs11089858, rs6000989, rs79839322 |
| 0.084 | rs2413493 |
| 0.109 | rs117991712 |
| 0.125 | rs760973, rs4384887, rs742395 |
| 0.131 | rs3026681 |
| 0.148 | rs3026688, rs880669 |
| 0.140 | rs713729, rs35706323 |
| 0.183 | rs9622722 |
| 0.188 | rs56111993, rs13053681 |
| 0.233 | rs2076369 |
| 0.238 | rs2018980, rs742396, rs2076370, rs738442, rs6000991 |
| 0.244 | rs738443, rs4821735 |
| 0.306 | rs10639701 |
| 0.392 | rs4821733 |
| 0.408 | rs4821734, rs5756893, rs34823615 |
| 0.416 | rs5756894 |
| 0.979 | rs3026682 |
| 1.000* | rs34385006, rs8138743, rs8139025, rs8142379, rs8142185, rs6519096, rs6519097, rs8142887, rs737662, rs5845364, rs13055597, rs3948, rs738441, rs6147619, rs10563532, rs3026685 |

| **Pairwise D' values** | **SNP** |
| --- | --- |
| 0.66 | rs9622722 |
| 0.826 | rs760973, rs4384887, rs742395 |
| 0.842 | rs10639701 |
| 0.852 | rs2413493 |
| 1 | rs11089858, rs6000989,  rs79839322, rs117991712,  rs3026681, rs3026688,  rs880669, rs713729,  rs35706323, rs56111993,  rs13053681, rs2076369,  rs2018980, rs742396,  rs2076370, rs738442,  rs6000991, rs738443,  rs4821735, rs4821733,  rs4821734, rs5756893,  rs34823615, rs5756894,  rs3026682, rs34385006,  rs8138743, rs8139025,  rs8142379, rs8142185,  rs6519096, rs6519097,  rs8142887, rs737662,  rs5845364, rs13055597,  rs3948, rs738441,  rs6147619, rs10563532,  rs3026685 |

**Supplementary Table S2.**Possible functional polymorphisms which have linkage disequilibrium with rs2076369: Pairwise r^2^ values and pairwise D' values for 22:38453652-38473651. Population: 1000GENOMES:phase_3:CHS

<http://grch37.ensembl.org/Homo_sapiens/Export/Output/Location?_format=HTML;db=core;output=ld;pop1=1000GENOMES:phase_3:CHS;r=22:38453652-38473651;v=rs2076369;vdb=variation;vf=1478380>.

| **Pairwise r^2^ values** | **SNP** |
| --- | --- |
| 0.057 | rs148328699 |
| 0.063 | rs79839322, rs6000989 |
| 0.104 | rs184204636 |
| 0.110 | rs713729, rs35706323 |
| 0.117 | rs3026688, rs880669 |
| 0.148 | rs56111993, rs13053681 |
| 0.233 | rs13055597, rs3952, rs3948, rs738441, rs6147619, rs10563532, rs3026685 |
| 0.258 | rs5756898 |
| 0.516 | rs760975 |
| 0.539 | rs10639701 |
| 0.956 | rs738443, rs4821735 |
| 0.978 | rs738442, rs6000991, rs2076370 |

| **Pairwise D' values** | **SNP** |
| --- | --- |
| 0.925 | rs5756898 |
| 1 | rs148328699, rs79839322, rs6000989,  rs184204636, rs713729, rs35706323,  rs3026688, rs880669, rs56111993, rs13053681, rs13055597, rs3952, rs3948, rs738441, rs6147619, rs10563532, rs3026685, rs760975, rs10639701,  rs738443, rs4821735, rs738442, rs6000991, rs2076370 |
